# 1-Hydroxy­ethyl-2-methyl-5-nitro­imidazolium 3-carb­oxy-4-hydroxy­benzene­sulfonate

**DOI:** 10.1107/S1600536808018643

**Published:** 2008-06-25

**Authors:** Bo Yang

**Affiliations:** a Wuhan Grand Pharmaceutical Group Co. Ltd, No. 5 Gu Tian Road, Wuhan 430035, People’s Republic of China

## Abstract

Cocrystallization of 1-hydroxy­ethyl-2-methyl-5-nitroimidazole (metronidazole) and 5-sulfosalicylic acid (5-H_2_SSA) from methanol solution yields the title salt, C_6_H_10_N_3_O_3_^+^·C_7_H_5_O_6_S^−^. In the crystal structure, the ions are linked by a combination of inter­molecular O—H⋯O, N—H⋯O and C—H⋯O hydrogen bonds, forming a three-dimensional framework. The hydroxyl group of the cation is disordered over two sites in a 0.860 (4):0.140 (4) ratio.

## Related literature

For related literature, see: Athar *et al.* (2005 ▶); Bharti *et al.* (2002 ▶); Castelli *et al.* (2000 ▶); Cohen-Jonathan *et al.* (2001 ▶); Crozet *et al.* (2002 ▶); Galván-Tejada *et al.* (2002 ▶); Hodgkiss (1998 ▶); Kennedy *et al.* (2006 ▶); Meng *et al.* (2007 ▶); Skupin *et al.* (1997 ▶); Wu *et al.* (2003 ▶).
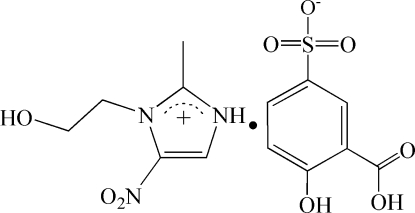


## Experimental

### 

#### Crystal data

C_6_H_10_N_3_O_3_^+^·C_7_H_5_O_6_S^−^
*M* *_r_* = 389.34
Monoclinic, 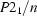

*a* = 8.8438 (3) Å
*b* = 13.0249 (4) Å
*c* = 14.148 (5) Å
β = 100.413 (1)°
*V* = 1602.9 (6) Å^3^
*Z* = 4
Mo *K*α radiation
μ = 0.26 mm^−1^
*T* = 294 (2) K
0.35 × 0.26 × 0.20 mm

#### Data collection

Bruker SMART APEX CCD area-detector diffractometer
Absorption correction: multi-scan (*SADABS*; Sheldrick, 1997 ▶) *T*_min_ = 0.904, *T*_max_ = 0.950
17399 measured reflections
3503 independent reflections
3156 reflections with *I* > 2σ(*I*)
*R*_int_ = 0.020

#### Refinement

*R*[*F* ^2^ > 2σ(*F* ^2^)] = 0.042
*wR*(*F* ^2^) = 0.118
*S* = 1.06
3503 reflections
262 parameters
4 restraints
H atoms treated by a mixture of independent and constrained refinement
Δρ_max_ = 0.32 e Å^−3^
Δρ_min_ = −0.30 e Å^−3^

### 

Data collection: *SMART* (Bruker, 2001 ▶); cell refinement: *SAINT* (Bruker, 2001 ▶); data reduction: *SAINT*; program(s) used to solve structure: *SHELXS97* (Sheldrick, 2008 ▶); program(s) used to refine structure: *SHELXL97* (Sheldrick, 2008 ▶); molecular graphics: *PLATON* (Spek, 2003 ▶); software used to prepare material for publication: *PLATON*.

## Supplementary Material

Crystal structure: contains datablocks global, I. DOI: 10.1107/S1600536808018643/lh2643sup1.cif

Structure factors: contains datablocks I. DOI: 10.1107/S1600536808018643/lh2643Isup2.hkl

Additional supplementary materials:  crystallographic information; 3D view; checkCIF report

## Figures and Tables

**Table 1:** Hydrogen-bond geometry (Å, °)

*D*—H⋯*A*	*D*—H	H⋯*A*	*D*⋯*A*	*D*—H⋯*A*
O3—H3*A*⋯O2	0.83 (3)	1.86 (3)	2.618 (2)	151 (3)
N2—H2⋯O6	0.81 (2)	1.97 (2)	2.7557 (19)	163 (2)
C13—H13*D*⋯O7^i^	0.97	2.46	3.280 (3)	142
C11—H11*C*⋯O4^ii^	0.96	2.55	3.457 (3)	157
O9—H9*A*⋯O5^iii^	0.82 (1)	2.129 (13)	2.940 (2)	170 (4)
O9′—H9′⋯O2^iv^	0.82 (1)	2.258 (13)	2.830 (2)	127 (2)
O1—H1⋯O4^v^	0.86 (3)	1.73 (3)	2.5801 (19)	171 (3)

Symmetry codes: (i) 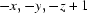; (ii) 
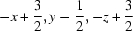
; (iii) 
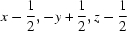
; (iv) 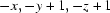; (v) 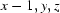.

**Fig. 1.**
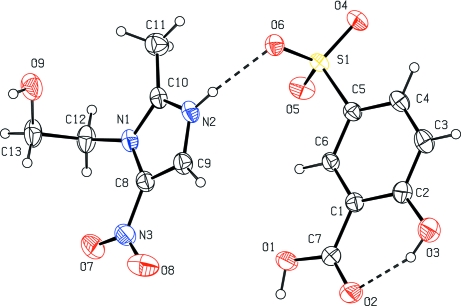
Molecular structure of (I), showing the atom-numbering scheme. Displacement ellipsoids are drawn at the 50% probability level. H-bonds are shown in dashed lines.

**Fig. 2.**
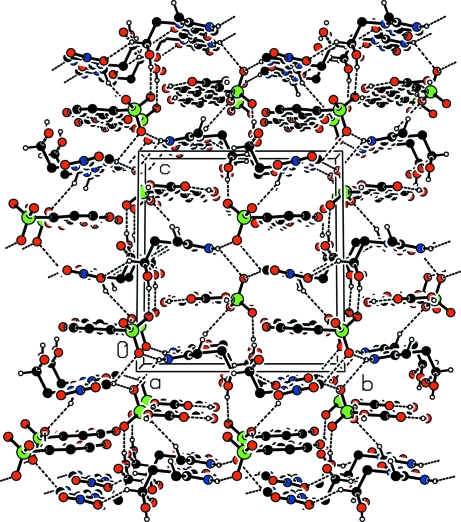
Part of the crystal structure of (I), showing the formation of the three-dimensional framework structure. Hydrogen bonds are shown as dashed lines. For the sake of clarity, H atoms not involved in the motif have been omitted from the drawing.

## supplementary crystallographic information

### Comment

1-Hydroxyethyl-2-methyl-5-nitrimidazole (metronidazole) is often used in the treatment of anaerobic protozoan and bacterial infections (Castelli *et al.*, 2000; Cohen-Jonathan *et al.*, 2001; Hodgkiss *et al.*, 1998). However, the low solubility in water makes its absorption in human body much less than expected. Recently, many efforts have been devoted to developing some new substitutes for the medicine, *i.e.* i) metal-organic coordination compounds (Kennedy *et al.*, 2006; Galván-Tejada *et al.*, 2002; Athar *et al.*, 2005; Bharti *et al.*, 2002; Wu *et al.*, 2003), ii) organic substitute derivatives (Crozet *et al.*, 2002, Skupin *et al.*, 1997) and iii) pharmaceutical co-crystals. In this paper, we report the 1:1 molecular adduct formed by metronidazole 5-sulfosalicylic acid (5-H_2_SSA), (I).

In (I), the H atom is transferred from the sulfonic acid group to the imidazole N atom (Fig.1) forming an 1:1 organic adduct, which is similar to the analogous organic adducts reported (Meng *et al.*, 2007). The hydroxyl O atom is disordered at two sites with occupancy being 0.86 (1)/0.14 (1) for the major and minor components, respectively.

In the crystal packing, the component ions are linked by a combination of O—H···O, N—H···O and C—H···O hydrogen bonds (Table 1), forming a three-dimensional network (Fig.2). There are no other interactions (*e.g.* C—H···π and π-π) observed in the crystal structure by using *PLATON* (Spek, 2003).

### Experimental

All the reagents and solvents were used as obtained without further purification. Equivalent molar amount of metronidazole and 5-sulfosalicylic acid dihydrate were dissolved in methanol (10 ml). The mixture was stirred for ten minutes at 300 K and then filtered. Block colorless crystals of (I) suitable for single-crystal X-ray diffraction analysis were grown by slow evaporation of the solution at the bottom of the vessel in two days.

### Refinement

H atoms bonded to C atoms were positioned geometrically [C–H = 0.93Å (aromatic), 0.97(methylene) and 0.96(methyl)] and refined in riding modes [*U*_iso_(H) = 1.2*U*_eq_(aromatic and methylene C) and 1.5*U*~eq~(methyl C]. H atoms bonded to N and O atoms were found in Fourier difference maps with the constraints of N—H = 0.86 (2) Å,*O*—H = 0.82 (2) Å, and *U*_iso_(H) = 1.2*U*_eq_(N) or 1.5*U*_eq_(O)]. The hydroxyl O atom is disordered at two sites with the occupancy being 0.86 (1):0.14 (1) for the major and minor components, respectively.

### Crystal data

**Table:**

C_6_H_10_N_3_O_3_^+^·C_7_H_5_O_6_S^–^	*F*_000_ = 808
*M**_r_* = 389.34	*D*_x_ = 1.613 Mg m^−^^3^
Monoclinic, *P*2_1_/*n*	Mo *K*α radiation λ = 0.71073 Å
Hall symbol: -P 2yn	Cell parameters from 9747 reflections
*a* = 8.8438 (3) Å	θ = 2.5–28.2º
*b* = 13.0249 (4) Å	µ = 0.26 mm^−^^1^
*c* = 14.148 (5) Å	*T* = 294 (2) K
β = 100.4130 (10)º	Block, colorless
*V* = 1602.9 (6) Å^3^	0.35 × 0.26 × 0.20 mm
*Z* = 4	

### Data collection

**Table:**

Bruker SMART APEX CCD area-detector diffractometer	3503 independent reflections
Radiation source: fine focus sealed Siemens Mo tube	3156 reflections with *I* > 2σ(*I*)
Monochromator: graphite	*R*_int_ = 0.020
*T* = 294(2) K	θ_max_ = 27.0º
0.3° wide ω exposures scans	θ_min_ = 2.1º
Absorption correction: multi-scan(SADABS; Sheldrick, 1997)	*h* = −11→11
*T*_min_ = 0.905, *T*_max_ = 0.950	*k* = −16→16
17399 measured reflections	*l* = −18→18

### Refinement

**Table:**

Refinement on *F*^2^	Secondary atom site location: difference Fourier map
Least-squares matrix: full	Hydrogen site location: inferred from neighbouring sites
*R*[*F*^2^ > 2σ(*F*^2^)] = 0.042	H atoms treated by a mixture of independent and constrained refinement
*wR*(*F*^2^) = 0.118	*w* = 1/[σ^2^(*F*_o_^2^) + (0.0695*P*)^2^ + 0.4716*P*] where *P* = (*F*_o_^2^ + 2*F*_c_^2^)/3
*S* = 1.07	(Δ/σ)_max_ < 0.001
3503 reflections	Δρ_max_ = 0.32 e Å^−^^3^
262 parameters	Δρ_min_ = −0.30 e Å^−^^3^
4 restraints	Extinction correction: none
Primary atom site location: structure-invariant direct methods	

### Special details

**Table:**

Geometry. All e.s.d.'s (except the e.s.d. in the dihedral angle between two l.s. planes) are estimated using the full covariance matrix. The cell e.s.d.'s are taken into account individually in the estimation of e.s.d.'s in distances, angles and torsion angles; correlations between e.s.d.'s in cell parameters are only used when they are defined by crystal symmetry. An approximate (isotropic) treatment of cell e.s.d.'s is used for estimating e.s.d.'s involving l.s. planes.
Refinement. Refinement of *F*^2^ against ALL reflections. The weighted *R*-factor *wR* and goodness of fit *S* are based on *F*^2^, conventional *R*-factors *R* are based on *F*, with *F* set to zero for negative *F*^2^. The threshold expression of *F*^2^ > σ(*F*^2^) is used only for calculating *R*-factors(gt) *etc*. and is not relevant to the choice of reflections for refinement. *R*-factors based on *F*^2^ are statistically about twice as large as those based on *F*, and *R*- factors based on ALL data will be even larger.

### Fractional atomic coordinates and isotropic or equivalent isotropic displacement parameters (Å^2^)

**Table:**

	*x*	*y*	*z*	*U*_iso_*/*U*_eq_	Occ. (<1)
C1	0.26714 (18)	0.68882 (13)	0.68052 (11)	0.0389 (3)	
C2	0.3234 (2)	0.78986 (13)	0.68903 (12)	0.0441 (4)	
C3	0.4814 (2)	0.80629 (14)	0.69525 (14)	0.0520 (4)	
H3	0.5198	0.8729	0.7007	0.062*	
C4	0.5807 (2)	0.72533 (14)	0.69348 (13)	0.0462 (4)	
H4	0.6851	0.7376	0.6965	0.055*	
C5	0.52498 (18)	0.62468 (13)	0.68713 (11)	0.0380 (3)	
C6	0.36914 (18)	0.60747 (13)	0.68103 (11)	0.0380 (3)	
H6	0.3320	0.5405	0.6772	0.046*	
C7	0.1012 (2)	0.67003 (14)	0.67435 (13)	0.0433 (4)	
C8	0.1649 (2)	0.27776 (14)	0.56001 (12)	0.0443 (4)	
C9	0.2283 (2)	0.36913 (14)	0.54592 (13)	0.0474 (4)	
H9	0.1782	0.4318	0.5342	0.057*	
C10	0.41167 (19)	0.25196 (13)	0.56990 (11)	0.0405 (4)	
C11	0.5662 (2)	0.20700 (17)	0.58081 (16)	0.0591 (5)	
H11A	0.6376	0.2584	0.5680	0.089*	
H11B	0.5650	0.1512	0.5363	0.089*	
H11C	0.5969	0.1820	0.6452	0.089*	
C12	0.2655 (3)	0.09157 (14)	0.58498 (13)	0.0546 (5)	
H12A	0.1935	0.0764	0.6273	0.065*	
H12B	0.3647	0.0633	0.6137	0.065*	
C13	0.2110 (3)	0.04186 (16)	0.48829 (16)	0.0625 (6)	
H13A	0.2000	−0.0315	0.4965	0.075*	0.860 (4)
H13B	0.1113	0.0694	0.4597	0.075*	0.860 (4)
H13C	0.2960	0.0404	0.4535	0.075*	0.140 (4)
H13D	0.1834	−0.0288	0.4987	0.075*	0.140 (4)
N1	0.27916 (17)	0.20437 (10)	0.57531 (10)	0.0415 (3)	
N2	0.38028 (18)	0.35061 (12)	0.55237 (11)	0.0441 (3)	
H2	0.447 (3)	0.3933 (19)	0.5509 (15)	0.055 (6)*	
N3	0.0052 (2)	0.26065 (16)	0.56153 (12)	0.0582 (4)	
O1	0.06220 (15)	0.57238 (10)	0.66817 (11)	0.0551 (4)	
H1	−0.029 (3)	0.567 (2)	0.6806 (19)	0.083*	
O2	0.00803 (16)	0.73868 (11)	0.67490 (13)	0.0644 (4)	
O3	0.23266 (19)	0.87220 (10)	0.69337 (12)	0.0612 (4)	
H3A	0.144 (4)	0.849 (3)	0.688 (2)	0.092*	
O4	0.80111 (14)	0.55223 (12)	0.72322 (11)	0.0577 (4)	
O5	0.59335 (15)	0.44064 (11)	0.74685 (10)	0.0552 (4)	
O6	0.62653 (14)	0.48356 (9)	0.58626 (9)	0.0447 (3)	
O7	−0.0358 (2)	0.17473 (15)	0.58045 (15)	0.0874 (6)	
O8	−0.0796 (2)	0.33520 (18)	0.54514 (15)	0.0865 (6)	
O9	0.3138 (2)	0.06004 (17)	0.42931 (13)	0.0725 (7)	0.860 (4)
H9A	0.260 (4)	0.065 (3)	0.3759 (14)	0.109*	0.860 (4)
O9'	0.1003 (12)	0.0853 (9)	0.4339 (7)	0.066 (4)	0.140 (4)
H9'	0.13 (3)	0.135 (12)	0.408 (15)	0.099*	0.140 (4)
S1	0.64580 (4)	0.51687 (3)	0.68609 (3)	0.03782 (14)	

### Atomic displacement parameters (Å^2^)

**Table:**

	*U*^11^	*U*^22^	*U*^33^	*U*^12^	*U*^13^	*U*^23^
C1	0.0401 (8)	0.0393 (8)	0.0371 (8)	−0.0030 (7)	0.0064 (6)	−0.0011 (6)
C2	0.0514 (10)	0.0369 (8)	0.0437 (9)	−0.0007 (7)	0.0078 (7)	0.0033 (7)
C3	0.0565 (11)	0.0377 (9)	0.0610 (11)	−0.0127 (8)	0.0084 (9)	0.0039 (8)
C4	0.0425 (9)	0.0445 (9)	0.0514 (10)	−0.0126 (7)	0.0077 (7)	0.0038 (7)
C5	0.0367 (8)	0.0395 (8)	0.0381 (8)	−0.0048 (6)	0.0070 (6)	0.0012 (6)
C6	0.0380 (8)	0.0358 (8)	0.0404 (8)	−0.0065 (6)	0.0075 (6)	−0.0027 (6)
C7	0.0412 (8)	0.0417 (9)	0.0472 (9)	0.0003 (7)	0.0088 (7)	−0.0034 (7)
C8	0.0451 (9)	0.0486 (10)	0.0391 (8)	−0.0103 (7)	0.0073 (7)	−0.0059 (7)
C9	0.0491 (9)	0.0401 (9)	0.0517 (10)	−0.0048 (7)	0.0058 (8)	−0.0027 (7)
C10	0.0464 (9)	0.0394 (8)	0.0343 (8)	−0.0087 (7)	0.0036 (6)	−0.0016 (6)
C11	0.0510 (10)	0.0617 (12)	0.0620 (12)	0.0035 (9)	0.0035 (9)	0.0017 (10)
C12	0.0836 (14)	0.0359 (9)	0.0447 (9)	−0.0151 (9)	0.0128 (9)	0.0021 (7)
C13	0.0888 (16)	0.0447 (10)	0.0561 (12)	−0.0255 (11)	0.0188 (11)	−0.0107 (9)
N1	0.0520 (8)	0.0365 (7)	0.0351 (7)	−0.0120 (6)	0.0060 (6)	−0.0017 (5)
N2	0.0465 (8)	0.0371 (7)	0.0480 (8)	−0.0133 (6)	0.0066 (6)	−0.0014 (6)
N3	0.0501 (9)	0.0760 (12)	0.0500 (9)	−0.0176 (9)	0.0134 (7)	−0.0135 (8)
O1	0.0359 (6)	0.0428 (7)	0.0875 (10)	−0.0024 (5)	0.0135 (6)	−0.0028 (6)
O2	0.0482 (7)	0.0479 (8)	0.0985 (12)	0.0063 (6)	0.0172 (7)	−0.0104 (7)
O3	0.0614 (8)	0.0360 (7)	0.0852 (11)	0.0023 (6)	0.0104 (8)	0.0021 (7)
O4	0.0333 (6)	0.0637 (9)	0.0728 (9)	−0.0072 (6)	0.0014 (6)	−0.0126 (7)
O5	0.0534 (7)	0.0544 (8)	0.0578 (8)	0.0007 (6)	0.0105 (6)	0.0214 (6)
O6	0.0486 (7)	0.0396 (6)	0.0456 (7)	−0.0045 (5)	0.0079 (5)	−0.0006 (5)
O7	0.0759 (11)	0.0821 (12)	0.1140 (15)	−0.0409 (10)	0.0429 (10)	−0.0266 (11)
O8	0.0517 (9)	0.1110 (16)	0.0978 (13)	0.0069 (10)	0.0161 (9)	0.0071 (11)
O9	0.0805 (13)	0.0892 (14)	0.0503 (10)	−0.0317 (11)	0.0186 (9)	−0.0189 (9)
O9'	0.082 (8)	0.066 (7)	0.047 (6)	0.001 (6)	0.006 (5)	−0.013 (5)
S1	0.0307 (2)	0.0400 (2)	0.0417 (2)	−0.00536 (14)	0.00351 (15)	0.00333 (15)

### Geometric parameters (Å, °)

**Table:**

C1—C6	1.391 (2)	C11—H11B	0.9600
C1—C2	1.405 (2)	C11—H11C	0.9600
C1—C7	1.475 (2)	C12—N1	1.482 (2)
C2—O3	1.348 (2)	C12—C13	1.512 (3)
C2—C3	1.400 (3)	C12—H12A	0.9700
C3—C4	1.376 (3)	C12—H12B	0.9700
C3—H3	0.9300	C13—O9'	1.265 (8)
C4—C5	1.398 (2)	C13—O9	1.361 (3)
C4—H4	0.9300	C13—H13A	0.9700
C5—C6	1.384 (2)	C13—H13B	0.9700
C5—S1	1.7662 (17)	C13—H13C	0.9700
C6—H6	0.9300	C13—H13D	0.9700
C7—O2	1.217 (2)	N2—H2	0.81 (2)
C7—O1	1.317 (2)	N3—O7	1.221 (3)
C8—C9	1.346 (2)	N3—O8	1.223 (3)
C8—N1	1.379 (2)	O1—H1	0.86 (3)
C8—N3	1.434 (2)	O3—H3A	0.83 (3)
C9—N2	1.352 (2)	O4—S1	1.4539 (12)
C9—H9	0.9300	O5—S1	1.4437 (13)
C10—N2	1.328 (2)	O6—S1	1.4579 (14)
C10—N1	1.340 (2)	O9—H13C	0.4764
C10—C11	1.469 (3)	O9—H9A	0.82 (1)
C11—H11A	0.9600	O9'—H9'	0.82 (1)
			
C6—C1—C2	119.60 (15)	C13—C12—H12B	109.4
C6—C1—C7	120.76 (15)	H12A—C12—H12B	108.0
C2—C1—C7	119.61 (15)	O9'—C13—O9	94.5 (6)
O3—C2—C3	118.11 (16)	O9'—C13—C12	116.4 (5)
O3—C2—C1	123.01 (16)	O9—C13—C12	109.93 (18)
C3—C2—C1	118.87 (16)	O9'—C13—H13A	115.5
C4—C3—C2	120.97 (16)	O9—C13—H13A	109.7
C4—C3—H3	119.5	C12—C13—H13A	109.7
C2—C3—H3	119.5	O9—C13—H13B	109.7
C3—C4—C5	120.09 (16)	C12—C13—H13B	109.7
C3—C4—H4	120.0	H13A—C13—H13B	108.2
C5—C4—H4	120.0	O9'—C13—H13C	106.6
C6—C5—C4	119.46 (16)	C12—C13—H13C	108.5
C6—C5—S1	117.90 (12)	H13A—C13—H13C	98.3
C4—C5—S1	122.63 (13)	H13B—C13—H13C	121.6
C5—C6—C1	120.98 (15)	O9'—C13—H13D	109.2
C5—C6—H6	119.5	O9—C13—H13D	118.2
C1—C6—H6	119.5	C12—C13—H13D	108.4
O2—C7—O1	122.75 (17)	H13B—C13—H13D	100.4
O2—C7—C1	123.05 (17)	H13C—C13—H13D	107.4
O1—C7—C1	114.20 (15)	C10—N1—C8	107.09 (14)
C9—C8—N1	108.85 (15)	C10—N1—C12	123.38 (16)
C9—C8—N3	125.28 (18)	C8—N1—C12	129.19 (16)
N1—C8—N3	125.85 (17)	C10—N2—C9	110.91 (15)
C8—C9—N2	105.54 (16)	C10—N2—H2	122.7 (16)
C8—C9—H9	127.2	C9—N2—H2	126.1 (16)
N2—C9—H9	127.2	O7—N3—O8	124.99 (19)
N2—C10—N1	107.62 (16)	O7—N3—C8	118.5 (2)
N2—C10—C11	124.35 (17)	O8—N3—C8	116.46 (19)
N1—C10—C11	128.03 (17)	C7—O1—H1	108 (2)
C10—C11—H11A	109.5	C2—O3—H3A	105 (2)
C10—C11—H11B	109.5	C13—O9—H9A	104 (3)
H11A—C11—H11B	109.5	H13C—O9—H9A	119.2
C10—C11—H11C	109.5	C13—O9'—H9'	109 (10)
H11A—C11—H11C	109.5	O5—S1—O4	112.76 (9)
H11B—C11—H11C	109.5	O5—S1—O6	112.23 (8)
N1—C12—C13	111.03 (16)	O4—S1—O6	112.44 (8)
N1—C12—H12A	109.4	O5—S1—C5	106.37 (8)
C13—C12—H12A	109.4	O4—S1—C5	106.18 (8)
N1—C12—H12B	109.4	O6—S1—C5	106.24 (7)
			
C6—C1—C2—O3	−176.99 (16)	C11—C10—N1—C8	−179.79 (17)
C7—C1—C2—O3	1.0 (3)	N2—C10—N1—C12	174.03 (15)
C6—C1—C2—C3	1.7 (2)	C11—C10—N1—C12	−6.0 (3)
C7—C1—C2—C3	179.71 (16)	C9—C8—N1—C10	−0.29 (19)
O3—C2—C3—C4	178.52 (17)	N3—C8—N1—C10	−178.63 (16)
C1—C2—C3—C4	−0.2 (3)	C9—C8—N1—C12	−173.61 (17)
C2—C3—C4—C5	−1.2 (3)	N3—C8—N1—C12	8.0 (3)
C3—C4—C5—C6	1.1 (3)	C13—C12—N1—C10	−96.5 (2)
C3—C4—C5—S1	−179.15 (14)	C13—C12—N1—C8	75.8 (2)
C4—C5—C6—C1	0.4 (2)	N1—C10—N2—C9	−0.10 (19)
S1—C5—C6—C1	−179.38 (12)	C11—C10—N2—C9	179.92 (17)
C2—C1—C6—C5	−1.8 (2)	C8—C9—N2—C10	−0.1 (2)
C7—C1—C6—C5	−179.78 (15)	C9—C8—N3—O7	−175.94 (19)
C6—C1—C7—O2	179.04 (18)	N1—C8—N3—O7	2.1 (3)
C2—C1—C7—O2	1.1 (3)	C9—C8—N3—O8	2.8 (3)
C6—C1—C7—O1	−1.0 (2)	N1—C8—N3—O8	−179.15 (17)
C2—C1—C7—O1	−178.93 (16)	C6—C5—S1—O5	−42.03 (15)
N1—C8—C9—N2	0.23 (19)	C4—C5—S1—O5	138.20 (15)
N3—C8—C9—N2	178.58 (16)	C6—C5—S1—O4	−162.37 (13)
N1—C12—C13—O9'	−44.9 (7)	C4—C5—S1—O4	17.86 (17)
N1—C12—C13—O9	61.0 (3)	C6—C5—S1—O6	77.73 (14)
N2—C10—N1—C8	0.23 (18)	C4—C5—S1—O6	−102.04 (15)

### Hydrogen-bond geometry (Å, °)

**Table:**

*D*—H···*A*	*D*—H	H···*A*	*D*···*A*	*D*—H···*A*
O3—H3A···O2	0.83 (3)	1.86 (3)	2.618 (2)	151 (3)
N2—H2···O6	0.81 (2)	1.97 (2)	2.7557 (19)	163 (2)
C13—H13D···O7^i^	0.97	2.46	3.280 (3)	142
C11—H11C···O4^ii^	0.96	2.55	3.457 (3)	157
O9—H9A···O5^iii^	0.82 (1)	2.129 (13)	2.940 (2)	170 (4)
O9'—H9'···O2^iv^	0.82 (1)	2.258 (13)	2.830 (2)	127 (2)
O1—H1···O4^v^	0.86 (3)	1.73 (3)	2.5801 (19)	171 (3)

Symmetry codes: (i) −*x*, −*y*, −*z*+1; (ii) −*x*+3/2, *y*−1/2, −*z*+3/2; (iii) *x*−1/2, −*y*+1/2, *z*−1/2; (iv) −*x*, −*y*+1, −*z*+1; (v) *x*−1, *y*, *z*.
